# Reduced sperm telomere length in individuals with varicocele is associated with reduced genomic integrity

**DOI:** 10.1038/s41598-019-40707-2

**Published:** 2019-03-13

**Authors:** Sahar Tahamtan, Marziyeh Tavalaee, Tayebeh Izadi, Nooshin Barikrow, Zahra Zakeri, Richard A. Lockshin, Homayoun Abbasi, Mohammad Hosseini Nasr- Esfahani

**Affiliations:** 1 Department of Reproductive Biotechnology, Reproductive Biomedicine Research Center, Royan Institute for Biotechnology, ACECR, Isfahan, Iran; 2 Department of Molecular and Cellular Sciences, Faculty of Advanced Sciences & Technology, Pharmaceutical Sciences Branch, Islamic Azad University, (IAUPS), Tehran, Iran; 3 Department of Cellular Biotechnology, Cell Science Research Center, Royan Institute for Biotechnology, ACECR, Isfahan, Iran; 4 Department of Biology, Queens College and Graduate Center of the City University of New York, Flushing, New York USA; 5 Department of Biological Sciences, St. John’s University, Jamaica, New York USA; 6 Isfahan Fertility and Infertility Center, Isfahan, Iran

## Abstract

Varicocele, defined as enlarged varicose veins in the scrotum, is the most common identifiable cause of male infertility. There are significant correlations between oxidative stress and varicocele-related infertility due to testicular hyperthermia, which can result in low sperm function. In addition, recent excessive oxidative stress can affect sperm telomere length and integrity of sperm DNA. Therefore, we assessed sperm telomere length as a potential marker of paternal genome integrity and leukocyte telomere length as an internal control (real-time PCR), along with sperm chromatin status (TUNEL and chromomycin A3 assay), and lipid peroxidation (Bodipy probe) in 18 infertile men with grade II or III varicocele, and 20 fertile men. Means of sperm parameters, sperm and leukocyte telomere length were significantly lower, while means of sperm DNA fragmentation, protamine deficiency, and lipid peroxidation were significantly higher in infertile men with varicocele compared to fertile men. Therefore, shortened telomere length in sperm and leukocytes is likely associated with increased oxidative stress related to the state of varicocele, which also accounts for increase in sperm DNA fragmentation. Thus, assessment of leukocyte telomere length could be taken as an indicator of antioxidant capacity in an individual, which also affects sperm function.

## Introduction

The term “varicocele” was initially suggested by the British surgeon T.B. Curling in 1843 to define venous dilatation of plexus pampiniform^1^. This phenomenon is one of the most important cause of male infertility in 35% of men with primary and 80% of men with secondary infertility, compared to 15% of general adult male population^2^. Increased testicular temperature, related to altered blood flow in the pampiniform plexus, results in testicular dysfunction with increased testicular oxidative stress appearing to be the main mediator^3,4^.

At the level of semen, testicular dysfunction manifests as decreased sperm concentration, motility and morphology while at the cellular level, it is associated with higher DNA fragmentation, apoptosis and reduced chromatin integrity compared to the fertile population^5–12^. In addition to these adverse effects, many cellular enzymatic processes are affected due to altered enzyme kinetic properties at increased temperature^13–16^.

Chromosomal stability or genomic integrity is highly dependent on telomere length at the ends of each chromosome. Telomeres are characterized as heterochromatic structures with noncoding hexanucleotide TTAGGG repeats that are maintained by telomerases. The protective function of telomeres is mediated through the nucleoprotein complex “shelterin” that binds to these hexameric repeats at the ends of each chromosome. Shelterin protects the chromosome from being attacked by exonucleases and prevents end-to-end fusion between chromosomes by inhibiting DNA damage response being activated as recognition of a double-strand break, and overall prevents DNA degradation, recombination, and DNA end fusions^17,18^.

The average telomere length was estimated between 5 to 10 kb in human somatic cells and 10–20 kb in germ cells^19^. Unlike somatic cells, in germ, stem and cancerous cells, the length of the telomere is preserved through cell divisions^20^. This difference has been mainly related to reduction or absence of telomerase activity in somatic cells compared to the germ, stem and cancerous cells. Reduced telomere length in germ-line cells is associated with aberrant meiotic synapsis, recombination, chromosomal segregation, chromosomal disjunction, gamete aneuploidy, apoptosis and developmental arrest post fertilization^21^. Indeed, in the human embryo, critically short telomere lengths are associated with increased rate of cytoplasmic fragmentation, reduced blastocyst formation and, thereby infertility^22^.

The telomere length is a complex trait that is determined by various factors including age, oxidative stress, age of the father at the time of conception, infection and social status like smoking^18^. Considering the destructive effects of oxidative stress on telomere length and the fact that in varicocele state, oxidative stress increases^23,24^, we aimed to compare sperm telomere length as a potential marker of paternal genome integrity, along with sperm chromatin status, and lipid peroxidation between infertile men with varicocele and fertile individuals. In addition, leukocyte telomere length was assessed as an internal control. The result of current study clearly shows that the varicocele state, in addition to its effects on the sperm DNA integrity, sperm lipid peroxidation level, protamine content and sperm parameters, might also affect telomere length.

## Results

### Comparison of age and semen parameters between fertile and infertile men with varicocele groups

Semen characteristics, male age and paternal age at conception (PAC) were compared between fertile individuals and infertile men with varicocele (Table 1). Paternal age was significantly higher in fertile individuals compared to infertile men with varicocele but no significant difference was found in PAC between the two groups (*p* > 0.05). Despite this result, considering the effect of age as confounding factor on sperm telomere length by some researchers, therefore, we evaluated the effect of age as a possible confounding factor on telomere length by regression model in infertile men with varicocele and fertile men. This result showed that age did not significantly affect the study parameters in this manuscript. Therefore, the significant values presented in this manuscript are based on data adjusted for age. The mean value of semen parameters including sperm count, concentration and motility were significantly lower in infertile men with varicocele compared to the fertile individuals, while the percentage of abnormal morphology was significantly higher in infertile men with varicocele (*p* < 0.05). Semen volume showed no significant difference between the two groups (*p* > 0.05).

**Table 1:** Comparison of male age and sperm parameters between infertile men with varicocele and fertile individuals.

Parameters	Varicocele	Fertile	*p*-value
Male age (year)	28.50 ± 5.52	41.38 ± 3.62	<0.001
Paternal age at conception (year)	28.39 ± 7.47	30.30 ± 7.04	0.47
Sperm concentration (10^6^/ml)	69.83 ± 12.53	135.1 ± 23.68	0.01
Sperm count (10^6^/ejaculate)	209 ± 36.6	423.46 ± 70.56	0.007
Sperm motility (%)	43.75 ± 4.07	62.03 ± 2.58	0.001
Abnormal sperm morphology (%)	97.66 ± 0.2	96.88 ± 0.2	0.04
Semen volume (ml)	2.98 ± 0.3	3.36 ± 0.38	0.427

### Comparison of sperm lipid peroxidation and chromatin status between fertile and infertile men with varicocele groups

**Figure 1:**
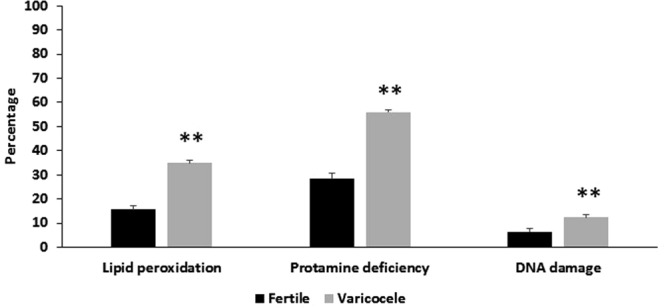
Comparison of mean percentage of sperm lipid peroxidation, protamine deficiency, and DNA damage between infertile men with varicocele and fertile individuals. *Shows *P* < 0.05 and **shows *P* < 0.001.

Comparison of percentage sperm lipid peroxidation (34.83 ± 3.4 vs. 15.54 ± 1.47; *p* < 0.001), protamine deficiency (55.89 ± 2.63 vs. 28.4 ± 2.33; *p* < 0.001) and DNA fragmentation (12.43 ± 1.23 vs. 6.52 ± 1.17; *p* = 0.003) between infertile men with varicocele and fertile individuals showed that the aforementioned parameters, as negative markers of fertility, were significantly higher in individuals with varicocele (Fig. 1).

### Comparison of sperm telomere length (STL), leucocyte telomere length (LTL) between fertile and infertile men with varicocele groups

**Figure 2:**
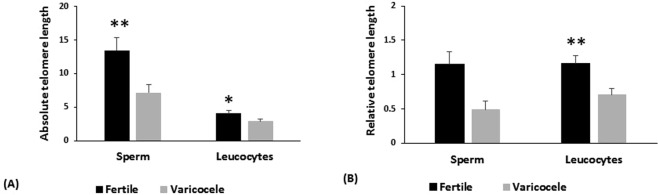
Comparison of (**A**) absolute and (**B**) relative telomere length in sperm and leucocyte between fertile individuals and infertile men with varicocele. *Shows *P* < 0.05 and **shows *P* < 0.01.

In this study, telomere length in sperm and leucocyte were evaluated by two methods; relative and absolute (Fig. 2). Means of absolute (7.17 ± 1.18 vs. 13.44 ± 1.87; *p* = 0.006) and relative (0.48 ± 0.12 vs. 1.14 ± 0.18; *p* = 0.004) telomere length in sperm were significantly lower in infertile men with varicocele compared to fertile individuals. Similar to telomere length in sperm, means of relative (0.71 ± 0.08 vs. 1.16 ± 0.11; *p* = 0.003) and absolute (2.92 ± 0.3 vs. 4.06 ± 0.44; *p* = 0.03) telomere length in leucocytes in infertile men with varicocele were also significantly lower than fertile individuals.

### Correlation between, male age, sperm parameters, sperm and leucocyte telomere length, sperm lipid peroxidation, and chromatin status

In this study, a positive significant correlation was observed between absolute sperm telomere length and absolute leucocyte telomere length (*r* = 0.396; *p* = 0.03) and negative significant correlations were observed between absolute sperm telomere length with percentage of DNA fragmentation (*r* = −0.401; *p* = 0.03), protamine deficiency (*r* = −0.438; *p* = 0.02) and sperm lipid peroxidation (*r* = −0.39; *p = *0.04). Similarly, absolute leucocyte telomere length showed significant negative correlation with percentage of DNA fragmentation (*r* = −0.542; *p* < 0.001) and protamine deficiency (*r* = −0.401; *p* = 0.03). In addition, leukocyte telomere length significantly correlated with sperm concentration (*r* = 0.435, *p* = 0.01) and sperm count (*r* = 0.473; *p* < 0.001). A positive significant correlation was observed between absolute sperm telomere length and percentage of sperm motility (*r* = 0.376; *p* = 0.04).

Percentage of DNA fragmentation and protamine deficiency both showed negative significant correlations with sperm concentration and sperm count (*p* < 0.001). In addition, a very strong significant correlation was observed between the percentage of DNA fragmentation and protamine deficiency (*r* = 0.701; *p* < 0.001). Percentage of sperm lipid peroxidation also significantly negatively correlated with sperm count (*r* = −0.371; *p* = 0.04) and positively correlated with protamine deficiency (*r* = 0.552; *p* < 0.001) (Table 2). It is important to note that, when we assessed the correlation between aforementioned parameters with relative sperm and leucocyte telomere length, we obtained similar results as with absolute values. Therefore, to make the results concise, we presented only the correlations with absolute telomere length.

**Table 2:** Shows the correlation among sperm parameters absolute sperm telomere length (STL), absolute leucocyte telomere length (LTL), sperm lipid peroxidation, protamine deficiency and DNA fragmentation.

Parameters	STL	LTL	DNA fragmentation	Protamine deficiency	Lipid peroxidation
Absolute sperm telomere length (STL)	1	0.396^*^	−0.401^*^	−0.438^*^	−0.390^*^
Absolute leucocyte telomere length (LTL)	0.396^*^	1	−0.542^**^	−0.401^*^	−0.213
Sperm concentration (10^6^/ml)	0.248	0.435^*^	−0.552^**^	−0.646^**^	−0.332
Sperm count (10^6^/ejaculate)	0.359	0.473^**^	−0.544^**^	−0.729^**^	−0.371^*^
Sperm motility (%)	0.376^*^	0.175	−0.261	−0.327	−0.331
Abnormal sperm morphology (%)	−0.298	−0.245	0.254	0.339	0.322
DNA fragmentation (%)	−0.401^*^	−0.542^**^	1	0.701^**^	0.260
Protamine deficiency (%)	−0.438^*^	−0.401^*^	0.701^**^	1	0.552^**^

**P < 0.001 and *P < 0.05.

## Discussion

Telomere length plays an important role in the maintenance of chromosomal stability and genomic integrity^25^. Therefore, researchers working in different fields have assessed the role of telomere length in aging-associated diseases, cancer and tumorigenesis, stem cell, coronary artery, cardiovascular disease and reproduction. In the latter field, they have assessed telomere length in oocyte, embryo, and sperm^18,20,26^. Some researchers believe that telomere length in human spermatozoa is unrelated to sperm parameters and DNA integrity. In this regard, Turner and Hartshorne stated that “our results lend weight to the idea that sperm telomere length is not crucial for male fertility, because telomere length resetting occurs in the embryo after fertilization. We propose that the oocyte probably modifies sperm telomere DNA, by a recombination based mechanism, before *de novo* telomerase transcription increases telomere length towards the blastocyst stage”^25,27–30^. In contrast, numerous studies suggest that sperm count, concentration, motility, and morphology show significant association with telomere length^25,31^. This disagreement could be related to confounding factors affecting both telomere length and infertility including age, oxidative stress, socioeconomic status, body mass index and genetic background^18^. Therefore, in this study, for the first time, the mean telomere lengths in infertile men with varicocele, which commonly presents with high oxidative stress, were compared to fertile individuals^24,32^.

In accordance with the literature, the results of this study also reveal that semen parameters of infertile men with varicocele showed significantly lower quality compared to fertile individuals, despite the fact that infertile men with varicocele were younger than the fertile individuals^5,9,10,33^. In addition, assessment of three important sperm functional parameters in these individuals revealed that degree of lipid peroxidation, protamine deficiency, and DNA damage were significantly higher in infertile men with varicocele. These observations are consistent with previous reports in the literature^7,23,34–36^. In this study, we assessed lipid peroxidation as the main marker of oxidative stress using Bodipy probe in infertile men with varicocele. We show that a high level of lipid peroxidation correlates with shortened sperm telomere length (Table 2).

Assessment of sperm and leucocyte telomere length by both absolute and relative methods show that for both cell types, the telomere length was significantly lower in infertile men with varicocele than fertile individuals. Comparison paternal age at conception revealed no difference between the two groups; however, mean age in fertile group was significantly higher than infertile men with varicocele. We initially thought that longer telomere length in fertile individuals is likely to be related to age, but assessment of telomere length in leucocyte revealed that in infertile men with varicocele, their leucocyte telomere length is also shorter than fertile men, despite their younger age, indicating that reduced telomere length in these individuals is likely to be independent of age and parental age at conception and is more likely to be an inherent problem in these individuals. Whether this inherent problem is a causative or associative factor with state of varicocele remains to be determined. Indeed, it has been shown that oxidative stress results in reduction of leucocyte telomere length. Antioxidant therapy can potentially decrease telomere attrition^37^.

Oxidative stress has been shown to affect the telomere length^18^. Indeed, we showed that both lipid peroxidation and DNA fragmentation, related to oxidative stress, are higher in these individuals. Increased oxidative stress at testicular level is a well-established fact in testes of infertile men with varicocele and may account for reduced telomere length in individuals with varicocele, but whether such an effect at somatic level could account for decreased telomere length in leucocytes remains to be determined^38,39^. The observed correlation between sperm and leucocyte telomere length may extend the support of this hypothesis. Therefore, the imbalance between ROS production and antioxidant capacity due to high percentage of abnormal sperm morphology, and number of leucocytes in infertile men with varicocele can induce oxidative stress and may account for decrease of both sperm and leucocyte telomere length^15,32^. Another possibility that may account for shortening of telomere length in sperm of infertile men with varicocele could be reduction of telomerase activity in testis. Indeed, concentration and activity of many testicular enzymes in infertile men with varicocele have reduced, possibly due to testicular hyperthermia, including: Topoisomerase I, phospholipase C zeta, acrosin activity, and apurinic/apyrimidinic endonuclease^11,13,40,41^.

It was also interesting to note that we observed significant negative correlations between telomere lengths of sperm and leukocytes as well as between DNA fragmentation and protamine deficiency. Unlike sperm telomere length, significant correlations were observed between sperm count and concentration with leukocyte telomere length. These results indicate that reduced telomere length renders sperm prone to loss of chromatin integrity, assessed as protamine deficiency or DNA fragmentation, or vice versa. This interaction may underlie the role of reduced antioxidant capacity at both testicular and somatic levels, which may render sperm prone to chromatin immaturity, increased DNA fragmentation, and reduced telomere length. Since we did not measure the antioxidant capacity at testicular and somatic levels, this hypothesis requires future verification. But our result reveals the potential of leukocyte telomere length as a biomarker for infertility.

In addition, significant correlations were observed between sperm motility (positive) and lipid peroxidation (negative) with sperm telomere length but not with leukocyte telomere length. These results indicate the importance of assessment of ROS at testicular level, which may be different from plasma levels. These observations are consistent with an *in vitro* study that showed that exposure of transcriptionally and translationally silent spermatozoa to H_2_O_2_ reduced telomere length, possibly directly, independent of reduced telomerase activity. This argument was justified by observation of oxidation of three guanine repeats in telomere repetitive sequences to 8-oxo-guanine^42^. In this study, the correlations observed among DNA fragmentation, lipid peroxidation and protamine deficiency are consistent with literature, suggesting that protamine deficiency and sperm immaturity render sperm prone to both DNA fragmentation and lipid peroxidation due to relaxation of chromatin and retention of extra cytoplasm, respectively^23,33^.

In conclusion, the result of this study shows that mean sperm telomere length in infertile men with varicocele is shorter than in fertile individuals and this is likely associated with increased oxidative stress correlated with the state of varicocele and which also accounts for increase sperm DNA fragmentation in these individuals. Further studies are needed to confirm these correlations. Furthermore, significant correlations were observed between leukocyte telomere length and semen parameters, sperm DNA fragmentation, and protamine deficiency. Therefore, assessment of leukocyte telomere length could be taken as indicator of antioxidant capacity in an individual that also reflects its effect on sperm function. Therefore, antioxidant therapy alone and/or along with varicocelectomy can improve fertility potential in infertile men with varicocele, which is in agreement with literature.

## Materials and Methods

### Participants

Following approval of the study by Royan Ethical Committee, semen samples and blood were collected from 20 fertile individuals referred for family balancing and 18 men with varicocele (II & III grade) referred to Isfahan Fertility and Infertility Center. Informed written consent was obtained from each participant. Infertile men with varicocele that (1) had other infertility related diseases, such as genital infection, hypogonadism, Klinefelter’s syndrome, testicular size discrepancy, anatomical disorders, abnormal hormonal profile, grade I varicocele, recurrent varicocele, azoospermia, previous history of scrotal trauma or surgery, and/or (2) were exposed to occupational agents and factors degrading male fertility, such as pesticides, solvents, heat, radiation, excessive alcohol, drug consumption, and/or (3) had other factors affecting seminal ROS level such as leukocytospermia, age >40 years, were excluded from this study. The diagnosis of varicocele was made by clinical examination and confirmed by color Doppler analysis. Clinical varicocele severity was graded according to the criteria of Dubin and Amelar RD^43^.

### Sperm preparation

Standard semen analysis was carried out according to World Health Organization protocol and assessments were carried out by one trained individual^44^. An aliquot of semen sample was used for assessment of sperm motility, concentration, and morphology by using computer-aided sperm analysis (CASA) and the remaining sample was used for assessment of protamine deficiency, DNA fragmentation, sperm lipid peroxidation, and sperm telomere lengths by Chromomycin A3 staining, TUNEL assay, Bodipy probe, and real-time PCR, respectively.

### Evaluation of sperm protamine deficiency using CMA3 staining

Chromomycin A3 (CMA3) staining was carried out according to Nasr-Esfahani *et al*.^45^. Briefly, semen samples were washed with PBS and fixed in Carnoy’s solution [methanol: glacial acetic acid 3:1 (Merck, Germany)]. Then, two smears were prepared for each sample and stained with CMA3 solution [0.25 mg/ml in McIlvaine buffer (7 ml citric acid 0.1 M, 32.9 ml Na_2_HPO_4_ .7H_2_O, 0.2 M, pH 7.0, containing 10 Mm MgCl_2_)] for 20 min. Next step, slides were washed and mounted. Status of sperm protamine content for each sample was evaluated using an Olympus fluorescence microscope (BX51, Tokyo, Japan) with the appropriate filters (460–470 nm), and 500 sperm cells were counted. Percentage of spermatozoa with bright yellow staining, as protamine deficient spermatozoa, was reported for each sample^45^.

### Evaluation of sperm DNA fragmentation using TUNEL assay

DNA fragmentation was carried out according to Tavalaee *et al*. using a terminal deoxynucleotidyl transferase dUTP nick end labeling (TUNEL) assay^46^. Briefly, semen samples were washed with PBS and fixed in 4% methanol-free formaldehyde on slides for 30 min. Then slides were washed and permeabilized with 0.2% Triton X-100 in PBS for 5 min. Next, a commercial detection kit from Promega company was used for the detection of DNA fragmentation (Apoptosis Detection System Fluorescein, Promega, Mannheim, Germany), according to the manufacturer’s instructions. 500 spermatozoa were randomly selected and evaluated using an Olympus fluorescence microscope (BX51; Tokyo, Japan) with the appropriate filters (460–470 nm) at 100× magnification. The TUNEL negative spermatozoa fluoresced red (spermatozoon without fragmented DNA), whereas the TUNEL-positive spermatozoa fluoresced bright green (spermatozoon with fragmented DNA).

### Assessment of sperm lipid peroxidation using the Bodipy probe

In this study, level of sperm lipid peroxidation was assessed according to the protocol by Aitken *et al*.^47^. Briefly, Bodipy C11 loading BODIPYw 581/591 C11 (D3861, Molecular Probes) was added to 2 × 10^6^ spermatozoa at a final concentration of 5 mM. Then, tubes were incubated for 30 min at 37 °C and samples were then washed twice with PBS buffer. Percentage of lipid peroxidation in sperm or Bodipy positive spermatozoa was assessed using a FACSCalibur flow cytometer (Becton Dickinson, San Jose, CA, USA).

### DNA extraction and telomere length assay by quantitative real-time PCR

Genomic DNA was extracted from the remaining washed sperm and peripheral blood leukocytes using QIAamp DNA Mini Kit (QIAGEN, Milan, Italy) according to manufacturer’s recommendations. Sperm telomere length (STL) and leucocyte telomere length (LTL) were determined by real-time polymerase chain reaction (qRT-PCR) as previously described by Cawthon. Each sample was run in triplicate^48^. Telomere length was expressed as relative and/or absolute value. The relative telomere length was calculated by the telomere to single-copy gene (T/S) ratio, and expressed as 2^(−ΔΔCt)^. For assessment of absolute telomere length according to modified method by NathanJ O’Callaghan *et al*.^49^, two standard curves were created by serial dilutions of known amounts of oligomer standards (36B4 and telomere) in each reaction, and then data were expressed as kbp.

### Statistical analysis

All of the statistical analyses were carried out using the Statistical Program for Social Sciences (SPSS Inc., Version 11.0, Chicago, IL, USA). Data were expressed as standard error of the mean (means ± SEM), except age, which was reported as the standard deviation of means (means ± SDM). The Kolmogorov–Smirnov Z test was used to assess the normal data distribution. Independent sample t-test was used for comparison of variations between fertile and infertile men with varicocele groups. Pearson correlation was carried out to analyze the association between different parameters. *p* < 0.05 was considered statistically significant.

### Ethical approval

All procedures performed in this study involving human participants were in accordance with the ethical standards of the Royan Ethical Committee (Project NO: 96000088).

### Informed consent

Informed consent was obtained from all individual participants included in the study.
